# The challenge of risk assessment in the riddle of Chagas heart disease

**DOI:** 10.1590/0074-02760210172chgsb

**Published:** 2022-06-06

**Authors:** José Antonio Marin-Neto, Anis Rassi

**Affiliations:** 1 Universidade de São Paulo, Divisão de Cardiologia da Faculdade de Medicina de Ribeirão Preto, Unidade de Cardiologia Intervencionista; 2 Diretor Científico do Hospital do Coração Anis Rassi, Goiânia, GO, Brasil

Morais-Torres et al. recently provided us with an extensive review on several pending challenges the scientific community still faces in order to decipher the conundrum of diagnosis, prognosis and the adequate treatment of patients with chronic Chagas cardiomyopathy (CCC).^1^ Among other no less conspicuous aspects of their review, it is relevant to emphasise that it is right on prognosis that substantial advances have been made over the last two decades of continuous progress in the understanding of the clinical issues concerning the management of CCC patients. And the authors of that important review in the Memórias do Instituto Oswaldo Cruz, in fact, did not fail to mention several relevant clinical aspects of CCC that connote plausible prognostic information. However, in our view, some attributes and some issues pertaining to the various risk scores that have been developed to predict the most serious events that assail patients with CCC deserve further critical consideration.

When a risk score is developed for a specific sample population, a first and more simple approach to validate the findings is through the partition of the whole original sample into two sub-samples; a first one, usually comprising two thirds of the sample, is used for the development of the score; and the remaining one third is used as a validation sub-sample. However, using a random sample for model development and the remaining patients for validation (split sample validation) is a suboptimal form of internal validation. Better methods are cross-validation and bootstrap resampling, where samples are drawn with replacement from the development sample.

Another strategy of validation that assures the general applicability of the model was employed when a score for prediction of overall mortality was developed by Rassi A Jr et al., in a non-selected cohort of 424 successive patients followed by Professor Anis Rassi in Goiânia, during an average of 7.9 years.^2^ The same investigators simultaneously conducted an external validation using an independent sample of 153 patients from another institution, the Evandro Chagas Clinical Research Institute, Oswaldo Cruz Foundation, Rio de Janeiro.^2^ A few months after the publication of that seminal research, a second external validation of the Rassi score was performed by independent investigators from the Federal University of Minas Gerais, in Belo Horizonte, Brazil, through a letter to the editor of the New England Journal of Medicine, reporting on a third cohort of 158 patients followed for five years or more.^3^

The Rassi score comprises two demographic/clinical characteristics (male sex, and dyspnea graded by the New York Heart Association as class III or IV) and four additional variables that are defined using simple laboratory tests [low voltage QRS in the 12-lead EKG, cardiomegaly detected with chest X-rays, left ventricular (LV) dysfunction assessed with a transthoracic resting echocardiogram, and the occurrence of non-sustained ventricular tachycardia (NSVT) evaluated by 24-hour Holter]. The results related to prognostication of mortality obtained using the Rassi score fully corroborate what had been found by two systematic reviews of several observational studies, that clearly showed that the strongest and most consistently independent predictors of mortality in patients with established CCC were advanced NYHA class, cardiomegaly, regionally or globally impaired LV systolic function, and NSVT.^4^ ^,^ ^5^

There have been other studies aiming to predict death risk in CCC patients. Following a first report on the prognostic value of signal-averaged ECG in 184 Chagas disease (CD) patients followed during 74 months,^6^ another investigation in 100 patients followed for an average of 95 months, reported that in addition to depressed LV systolic function and NSVT, positive intraventricular electrical transients obtained by signal averaged ECG was an independent predictor of ventricular tachycardia and death in CD.^7^ Although the other variables and tests required for the calculation of the Rassi score were apparently available in these two studies, the investigators did not explore whether the new variables, the prolonged filtered QRS duration or the positive intraventricular electrical transients, would add any significant and independent prognostic meaning to the already validated six variables included in the landmark Rassi score development study.

Another attempt to develop a risk score in CCC was conducted by investigators from Rio de Janeiro, Brazil, now focusing specifically on sudden death, the most frequent mechanism of death in patients with CCC, regardless of the degree of myocardial involvement.^8^ Their cohort enrolled 373 CCC patients, followed for a mean period of 66 ± 44 months, during which 43 patients experienced sudden death. In this study again no external validation was reported, and of those variables included in the Rassi score, only severe LV dysfunction was independently identified as a sudden death risk marker, together with syncope, ventricular extrasystoles, QT-interval dispersion to compose the proposed score for prediction of sudden death risk.

Still another study assessed death risk of 1551 patients with CCC from Minas Gerais, Brazil, but instead of the already validated clinical and laboratory variables the investigators reported on high resting heart rate, abnormal QRS duration and an elevated value of NT-proBNP as significant prognostic factors.^9^ This approach would not require exams such as the echocardiogram or Holter, however its superiority over the variables obtainable with these methods remains to be demonstrated.

More recently, a study provided another external validation of the Rassi score in a cohort of 130 patients followed for a median of five years by investigators from the University of São Paulo, this time not only for prediction of all-cause mortality, but for assessment of risk related to the composite outcome of all-cause death, heart transplantation, anti-tachycardia pacing or appropriate shock from an implantable cardioverter-defibrillator, and aborted sudden cardiac death.^10^ In addition, myocardial fibrosis (MF) as detected in vivo by cardiac magnetic resonance (CMR), was an independent predictor of that study composite outcome. Even if in the main paper reporting on those findings the effect of incorporating the new information about the presence of MF was not described, in a subsequent letter to the editor the authors performed a post-hoc subgroup analysis of their data and suggested a potential role for detection of MF to re-stratify risk in CCC in those patients with lower risk by the Rassi score.^11^ These data corroborate previous findings from a prospective cohort of 140 patients with CCC followed by investigators from the University of São Paulo in Ribeirão Preto, for a median 34 months, reporting that MF detected using CMR was strongly associated with the combined risk of cardiovascular death and sustained ventricular tachycardia.^12^

The Rassi score, as originally developed, included echocardiographic detection of LV systolic dysfunction either global or only as regional wall motion impairment as an independent marker of poor prognosis. That despite normal global LV systolic function, a score index related to regional wall motion abnormalities could identify CCC patients at higher risk of the composite outcomes included in the BENEFIT study primary outcome was demonstrated through a prospective sub-study of 1508 patients followed for a mean of 5.4 years.^13^

Several other investigators have reported on various clinical and laboratory abnormalities or risk models with prognostic meaning in patients with CCC. It is relevant to emphasise that models without external validation correspond to the lowest level of evidence and are not indicated for use in clinical practice. Therefore, we recommend researchers to spend more energy, in order to validate and assess whether the new prognostic factor adds to risk detection as determined by the existing prognostic indices, instead of merely developing more models that will most likely never be incorporated into daily care. Also, it is relevant consider that for a prognostic score to guide therapy in the context of its risk prediction, the proposed therapeutic approach should ideally be validated through the performance of randomised clinical trials.

Comments on the article: Torres RM, Correia D, Nunes MCP, Dutra WO, Talvani A, Sousa AS, et al. Prognosis of chronic Chagas heart disease and other pending clinical challenges. Mem Inst Oswaldo Cruz. 2022; 117: e210172.
